# Giant fibrovascular polyp of the oesophagus: a case report and review of the literature

**DOI:** 10.1186/1752-1947-2-337

**Published:** 2008-10-28

**Authors:** Danai Chourmouzi, Antonios Drevelegas

**Affiliations:** 1 Department of Diagnostic Radiology, Interbalcan Medical Center, Gymnasiou 20, Panorama 55236, Thessaloniki, Greece; 2 Department of Diagnostic Radiology, Ahepa University Hospital, Nikis 10, Panorama 55236, Thessaloniki, Greece

## Abstract

**Introduction:**

We present a case of fibrovascular polyp, a rare submucosal tumour of the oesophagus that has been reported only sporadically in the literature. The biapproach for surgical removal of fibrovascular polyp has only been mentioned once in the literature.

**Case presentation:**

A 65-year-old Greek man presented with a 9-month history of gradually progressive intermittent dysphagia. Radiologic work-up with oesophagogram and computed tomography revealed a large, sausage-shaped intraluminal polyp extending from the level of the cervical oesophagus to the level of the upper body of the stomach. The diagnosis of giant fibrovascular polyp was made radiographically and confirmed by endoscopic biopsy. The polyp was removed using a biapproach surgical technique: pharyngotomy and subsequent gastrostomy.

**Conclusion:**

Fibrovascular polyp is a rare submucosal tumour. Proper treatment depends on accurate assessment of the origin, size, and vascularity of the pedicle and the size of the tumour. Choice of the appropriate surgical approach depends on the correct diagnosis, which can usually be indicated radiographically by the presence of a smooth, sausage-shaped defect with a discrete bulbous tip.

## Introduction

A fibrovascular polyp (FVP) is a rare, benign, intraluminal, submucosal tumour-like lesion, characterised by the development of pedunculated, intraluminal masses that can exhibit enormous intraluminal growth. These lesions are composed of loose or dense fibrous tissue, adipose tissue, and vascular structures and are covered by normal squamous epithelium. The most common location is the upper third of the oesophagus, near the cricopharyngeus. Dysphagia, vomiting, weight loss, and respiratory symptoms are the most frequent complaints. However, long pedunculated lesions can regurgitate into the pharynx or mouth and cause death from asphyxiation if the larynx is occluded [1]. We present a case of FVP associated with intermittent dysphagia.

## Case presentation

A 65-year-old Greek man presented with a 9-month history of gradually progressive intermittent dysphagia. He also reported significant weight loss but no haemetemesis or melaena. The rest of his medical history was not significant. No specific abnormality was revealed during the physical examination.

**Figure 1:**
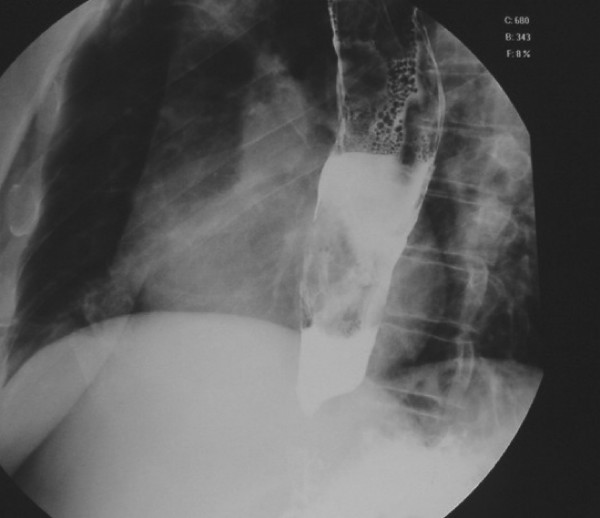
Barium oesophagogram showing dilatation of the entire oesophagus, multiple air bubbles, and filling defects.

**Figure 2:**
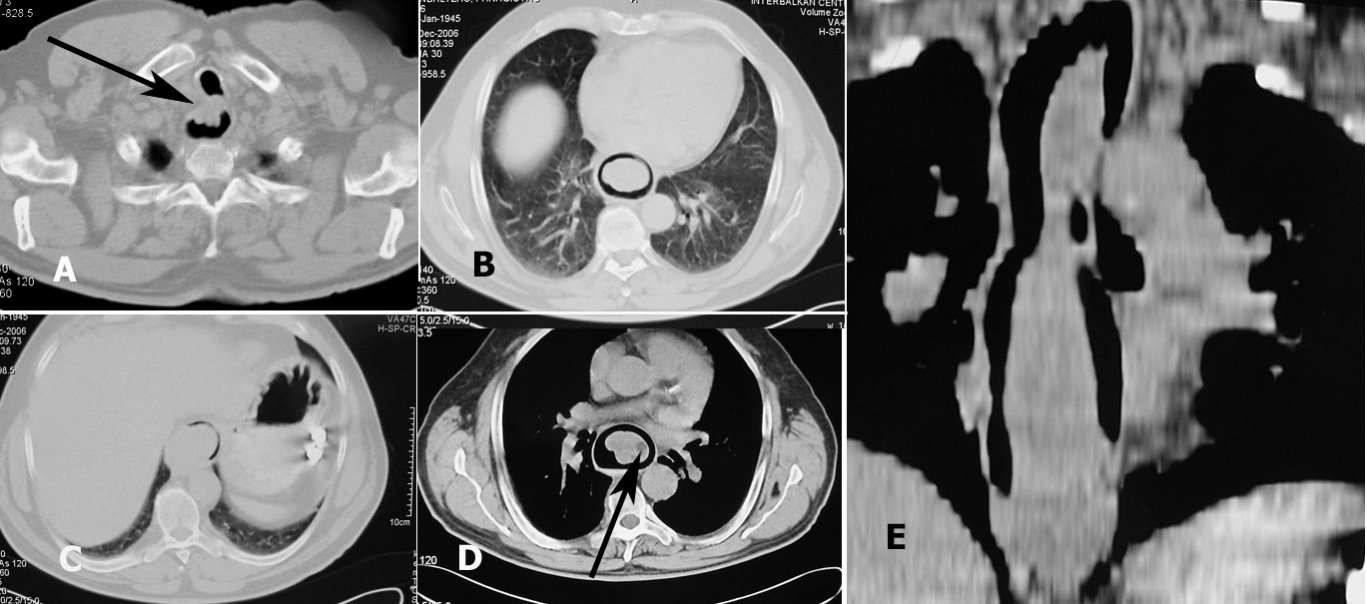
**Serial axial images of the chest computed tomography scan (a,b,c,d) from the level of upper oesophagus to the level of the stomach reveal a soft-tissue, large intraluminal lesion.** The polyp originates at the anterior wall of the cervical oesophagus (arrow in a). Axial computed tomography image (mediastinal window setting) shows an area of fat density (arrow in d). Reformatted coronal computed tomography image shows the entire length of the polyp (e).

Radiologic work-up with oesophagogram showed a dilated oesophagus, air bubbles with a mottled appearance, and contrast-filling defects from the cervical oesophagus to the upper body of the stomach (Figure 1). The computed tomography (CT) scan revealed a soft-tissue lesion in the oesophagus, extending from the level of the cervical oesophagus to the level of the upper body of the stomach (Figure 2a,b,c). The lesion appeared as a relatively smooth, sausage-shaped intraluminal mass with bulbous distal tip.

CT with multiplanar reformatting provided valuable information regarding the location of the lesion as well as the size and anatomical attachment proximally, which was pivotal in directing surgery (Figure 2d). The intraluminal polyp showed soft tissue densities and a central area with attenuation identical to that of fat (Figure 2e). Endoscopy revealed a smooth submucosal mass occluding the oesophageal lumen. Endoscopic ultrasound-guided fine-needle aspiration was performed and cytological examination revealed benign fibro-fatty elements. The lesion was diagnosed as a submucosal FVP of the oesophagus, originating from the cervical oesophagus.

**Figure 3:**
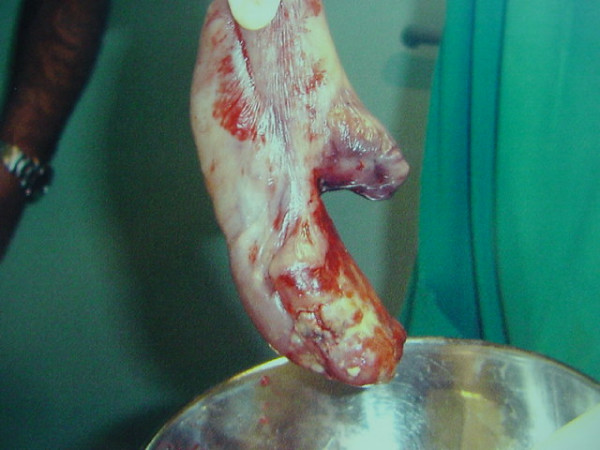
**Gross surgical specimen of the fibrovascular polyp.** The tumour was covered with a smooth, pinkish-grey mucosa similar to that of the normal oesophagus.

A biapproach surgical technique was selected. The origin of the pedicle was attached to the anterior wall of the hypopharynx. The broad base of the stalk was divided by performing cervical vertical oesophagostomy. However, the head of the polyp was too large to be removed through pharyngotomy; therefore, the entire polyp was removed via gastrostomy (Figure 3). The length of the tumour was 16 cm and histopathology revealed an oesophageal mucosa-covered polypoidal lesion composed of lymphocytes and plasma cells interspersed with fibroblasts and blood vessels. No hyperplasia of the mucosal epithelium was evident. The final diagnosis was FVP of the proximal oesophagus. The patient recovered uneventfully and was cured of his dysphagia.

## Discussion

FVPs are rare submucosal tumours of the oesophagus almost always originating from the cervical oesophagus; they are benign but potentially life-threatening lesions. In the past, these lesions have been variably classified as 'lipomas', 'fibromas', and 'fibrolipomatous' polyps [2-5]. They usually arise in the proximal oesophagus behind the cricoid cartilage, frequently from the upper oesophageal sphincter. These polyps usually originate as small mucosal tumours just below the cricopharyngeus muscle sphincter, then extend into the oesophageal lumen by the constant downward urge of both food and peristalsis, and they may reach into the stomach [6,7]. The incidence of these tumours is highest in middle-aged and elderly men, although some cases have occurred in children, infants, and women.

A FVP usually presents as a large, pedunculated lesion, and symptoms occur only once the polyp has become sufficiently large. Patients usually complain of dysphagia, substernal discomfort, and the sensation of a mass. Many cases of FVP have presented as regurgitated masses in the mouth, others have led to airway obstruction when the mass impacted on the larynx. Asphyxiation can result from impaction of the polyp in the glottis and is the most feared complication [8]. Histologically, the lesion is composed of variable admixtures of mature adipose tissue lobules, collagenous and sometimes myxoid tissue, and prominent vasculature (a mixture of muscular arteries, thin-walled veins, and capillaries), all surrounded by mature squamous epithelium. Malignant degeneration of FVP is thought to be extremely rare.

Unless regurgitated, the presence of FVP can be difficult to diagnose, and patients may die without a correct diagnosis. FVPs can sometimes be identified during chest radiography by the presence of a right-sided superior mediastinal mass, anterior tracheal bowing, or both. At oesophagography, the lesion usually appears as a smooth, expansile intraluminal mass that arises in the cervical oesophagus and extends into the thoracic oesophagus. On CT scan, FVPs containing abundant adipose tissue may appear as soft-tissue-attenuated lesions (abundant fibrovascular tissue), with a paucity of fat, that expand the lumen of the oesophagus.

Although most FVPs have an attachment site in the cervical oesophagus, barium studies often fail to demonstrate a proximal pedicle. Accurate diagnosis is best established with endoscopy, although this technique is not completely reliable for diagnosing FVP. The differential diagnosis for FVP includes achalasia, extrinsic masses that compress the oesophagus, giant coalescent air bubbles, and other polypoid intraluminal tumours including lymphoma, spindle cell carcinoma, malignant melanoma, and leiomyosarcoma. The correct diagnosis can usually be suggested radiographically by the presence of a smooth, sausage-shaped defect with a discrete bulbous tip [9].

Removal of these lesions is usually recommended because of the progressive and eventually debilitating nature of the symptoms and the small but known risk of asphyxiation and sudden death. The most common therapeutic approach is surgical cervical oesophagostomy with complete excision of the stalk. The location of the stalk and the vascularity makes surgical resection the preferred mode for removing these unusual polyps. Endoscopic resection is possible but is generally avoided because of the potential for haemorrhage from the feeding vessels in the stalk. However, complication-free endoscopic resection of large oesophageal giant FVPs has been reported.

Generally, small polyps less than 2 cm in diameter and with a thin pedicle can be removed by endoscopic ligation and electrocoagulation of the pedicle. Polyps larger than 8 cm long or those with a thick, richly vascularised pedicle should be removed by surgical excision, and usually through a cervical incision. When the head of the polyp is too large to be removed through pharyngotomy, removal via gastrostomy is recommended. We could identify only one report in the literature about the surgical resection of FVP using the biapproach [10]. A biapproach for surgical resection and removal of an FVP via pharyngotomy and gastrostomy is essential in cases of a large polyp that reaches into the stomach and has a bulbous distal tip.

## Conclusion

Giant FVP of the oesophagus is a very rare entity and few reports on this lesion exist in the literature. Diagnosis can be difficult for physicians who are unfamiliar with this type of tumour. The most common location is the upper third of the oesophagus, near the cricopharyngeus. Dysphagia, vomiting, weight loss, and respiratory symptoms are the most frequent complaints. However, long pedunculated lesions can regurgitate into the pharynx or mouth and cause death from asphyxiation if the larynx is occluded. The details of our case should raise awareness for both radiologists and clinical physicians. Oesophagogram and CT are essential when evaluating a patient with such symptoms.

## Competing interests

The authors declare that they have no competing interests.

## Authors' contributions

DC analysed and interpreted the patient data and was a major contributor in writing the manuscript. AD analysed the patient data and contributed in writing the manuscript. Both authors read and approved the final manuscript.

## Consent

Written informed consent was obtained from the patient for publication of this case report and any accompanying images. A copy of the written consent is available for review by the Editor-in-Chief of this journal.

## References

[B1] Palanivelu C Rangarajan M John SJ Annapoorni S Senthilkumar S A rare cause of intermittent dysphagia: giant fibrovascular polyp of the proximal esophagus J Coll Physicians Surg Pak 2007 17 1 51 52 17204222

[B2] Drenth J Wobbes T Bonenkamp J Nagengast F Recurrent esophageal fibrovascular polyps case history and review of the literature Dig Dis Sci 2002 47 2598 2604 10.1023/A:1020592900552 12452402

[B3] Lewin K Appelman H Rosai J, Sobin LH Mesenchymal tumors and tumor-like proliferations of the esophagus Tumors of the Esophagus and Stomach. Atlas of Tumor Pathology, 3rd series, fascicle 18 1996 Washington DC: Armed Forces Institute of Pathology 145 161

[B4] Wu MH Chuang CM Tseng YL Giant intraluminal polyp of the esophagus Hepatogastroenterology 1998 45 2115 2116 9951875

[B5] Carrick C Collins K Lee C Prahlow J Barnard J Sudden death due to asphyxia by esophageal polyp Am J Forensic Med Pathol 2005 26 275 281 10.1097/01.paf.0000178098.33597.de 16121086

[B6] Rees CJ Belafsky PC Giant fibrovascular polyp of the esophagus Ear Nose Throat J 2007 86 10 606 17990679

[B7] Kanaan S DeMeester TR Fibrovascular polyp of the esophagus requiring esophagectomy Dis Esophagus 2007 20 5 453 454 10.1111/j.1442-2050.2007.00710.x 17760662

[B8] Alobid I Vilaseca I Fernández J Bordas JM Giant fibrovascular polyp of the esophagus causing sudden dyspnea: endoscopic treatment Laryngoscope 2007 117 5 944 945 10.1097/MLG.0b013e318033310f 17473701

[B9] Ridge C Geoghegan T Govender P McDermontt R Torreggiani W Giant oesophageal fibrovascular polyp (2005:12b) Eur Radiol 2006 16 3 764 766 10.1007/s00330-005-0112-2 16470418

[B10] Hoseok I Kim JS Shim YM Giant fibrovascular polyp of the hypopharynx: surgical treatment with the biapproach J Korean Med Sci 2006 21 4 749 751 10.3346/jkms.2006.21.4.749 16891825 PMC2729903

